# Recent Developments in Pediatric Nephrology

**DOI:** 10.3390/jcm14051758

**Published:** 2025-03-05

**Authors:** Anna Wasilewska

**Affiliations:** Department of Paediatrics and Nephrology, Medical University of Białystok, 15-274 Białystok, Poland; anna.wasilewska@udsk.pl

## 1. Urinary Tract Infection and Vesicoureteral Reflux

Urinary tract infection (UTI) is a common bacterial infection in children that affects 1.7% of boys and 8.4% of girls before the age of 7 [1].

In 2021, new updated guidelines on how to treat a child with a UTI were published. As far as treatment is concerned, the use of second- or third-generation cephalosporins and amoxicillin with clavulanic acid in the treatment was maintained, but only with confirmed sensitivity. In children up to 3 months of age, a third-generation cephalosporin or a combination of ampicillin and aminoglycoside is recommended. Because of increasing antibiotic resistance, the guidelines introduce a provision for changing treatment to an antibiotic with the narrowest spectrum after receiving the result of the urine. In addition, the guidelines of the Polish Society of Paediatric Nephrology (PTNFDz) introduce a recommendation to treat acute pyelonephritis in pregnant adolescents in whom hospitalization and empirical therapy with second-generation intravenous cephalosporin are recommended. In case of pregnant adolescent the gynecologist consultation is recommended.

Very important recommendations concern asymptomatic bacteriuria. Urinalysis screening for asymptomatic bacteriuria and its treatment is not recommended. This also applies to patients with a catheter remaining in the bladder for up to 30 days. Urine culture should be performed before a potentially traumatizing instrumental procedure within the urinary tract, and if asymptomatic bacteriuria is found, two doses of a targeted antibiotic should be administered: before and after the procedure.

Significant changes also apply to the management of patients who are carriers of *Pseudomonas aeruginosa*. Since complete eradication of the pathogen is usually impossible, antibiotic therapy is not recommended unless there are clinical signs of a UTI. Bacteriuria and pyuria without UTI symptoms are not an indication for treatment. Treatment should apply to patients who are planning an instrumental urinary tract procedure or surgery.

The last part of the recommendations concerns the management of children after a UTI. One of the important aspects is antibacterial prevention. Because of the increasing resistance caused by prolonged antibiotic therapy, it is believed that the decision to use antibacterial prophylaxis should be made by a pediatric nephrologist after exhausting non-pharmacological methods. The decision on chronic prevention should be reviewed every 6 months.

Pharmacological prophylaxis of UTIs in children with prenatal suspicion of urinary tract defects is not normally recommended. However, the child should be monitored for a UTI until the urinary tract diagnosis is completed. It is recommended to use pharmacological prophylaxis for a UTI in children with vesicoureteral reflux of III–V degrees. Routine antimicrobial prophylaxis is not recommended to prevent catheter-related infections, either in the case of a single catheterization or if the catheter is left in the bladder for an extended period of time.

Another very important aspect is to determine the indications for nephrological diagnostics. Currently, ultrasound is recommended in all children up to 24 months of age after the diagnosis of the first episode of a UTI and in children >24 months of age after diagnosis of acute pyelonephritis, a UTI with an atypical course, or with risk factors for recurrence or with UTI recurrence [2].

Guidelines from the American Academy of Pediatrics and the Canadian Paediatric Society recommend kidney ultrasound in all children aged 2–24 months and <24 months after the first febrile UTI [3,4]. However, guidelines differ in other regions of the world. Some recommend performing an ultrasound of the kidneys after the first febrile UTI even after 24 months (e.g., up to 36 months or at any age) [5,6,7].

According to the UK National Institute for Health and Care Excellence (NICE) guidelines, kidney ultrasound is recommended for children <6 months and only for children aged 6 months to 16 years if a UTI is recurrent or atypical [8].

In 2023, a systematic review and meta-analysis were published to determine the indications for ultrasound after febrile UTI. The results of a study conducted in 9170 children showed that abnormalities will be found during an ultrasound of the kidneys in one in four–five (22%) children with the first febrile UTI, and in one in thirty-two (3%), these abnormalities will change the way they are clinically managed [9]. Of course, the optimal solution would be to perform this test in all children who have had a UTI, but both the costs and the availability of ultrasound in an outpatient setting should be considered.

The problem of indications for continuous antibacterial prophylaxis has been widely studied and discussed in recent years, especially in children with OPM. There are still no clear guidelines for its use in infants with OPM to prevent UTIs and potential long-term complications associated with kidney scarring. The controversy on this topic stems from the attempt to reduce the incidence of infections and the potential complications associated with them, on the one hand, and the risks associated with the emergence of multi-resistant strains and the negative impact of antibiotic therapy on the gut microbiota, on the other. Previous pediatric studies in UTI prevention have mainly focused on children with recurrent infections, mainly girls with associated bladder or bowel dysfunction [10,11]. In a large randomized trial (RCT), Randomized Intervention for Children with Vesicoureteral Reflux (RIVUR), in which children underwent randomization after the first or second UTI, >90% were female patients over a wide age range, and 80% had stage II or III VUR. It has been shown that, although long-term antibiotic prophylaxis was effective in preventing the recurrence of UTIs, it did not significantly affect kidney scarring, which raises doubts as to its usefulness [12].

A very important study from the point of view of dealing with a child with OPM is the PREDICT study, the results of which were published in 2023 [13]. It concerns the assessment of the effectiveness of continuous antibiotic prophylaxis in the prevention of UTIs in infants with OPM III, IV, or V degree. The results are very important from a clinical point of view for both pediatric nephrologists, pediatricians, and family medicine doctors. Using continuous antibacterial prophylaxis in this group of children for 24 months reduced the risk of UTI by almost 15%, but it should be emphasized that almost 65% of children with OPM III to V who did not receive any treatment did not develop a UTI. Continuous antibacterial prophylaxis was also not associated with the occurrence of fresh scarring in the kidneys, and it had no effect on eGFR at 24 months. Instead, an increased number of urine cultures with an increase in *Pseudomonas* and non-*E. coli bacteria* and increased antibiotic resistance were observed. There was no significant difference in the percentage of UTIs requiring hospitalization. In addition, the authors of the study believe that despite the defect appearing in the ultrasound examination, in the absence of a symptomatic UTI, there are no indications for invasive diagnostic procedures, especially voiding cystourethrography, as the result may not affect further management. Imaging tests should only be performed if congenital posterior urethral valves or ureterocele are suspected [13].

The results of the above study show that only 1/3 of untreated infants with stage III, IV, or V will develop a UTI within the first 2 years of life. The greatest benefits from continuous antibiotic prophylaxis were achieved by girls with stage IV and V VUR.

## 2. Hemolytic–Uremic Syndrome

In recent years, significant progress has been made in the pathogenesis and treatment of hemolytic–uremic syndrome (HUS).

### 2.1. STEC-HUS Treatment

Studies on the role of eculizumab in the treatment of HUS associated with Shiga toxin-producing Escherichia coli (STEC-HUS) infection are ongoing, but the results are inconclusive. There are reports of the effect of eculizumab on the increase in platelet counts and the reduction in central nervous system symptoms. Two placebo-controlled RCTs completed recruitment (ECUSTEC in the United Kingdom and ECULISHU in France), with preliminary results from the former not supporting clear evidence of the efficacy of eculizumab in STEC-HUS [14].

Currently, the treatment of STEC-HUS is still symptomatic. In severe cases of STEC-HUS, plasma exchange may also play a vital role in reducing circulating cytokines, Stx toxin, and von Willebrand factor multimolecular multimers, which contribute to endothelial damage, although there are few data to support its routine use [15].

### 2.2. Treatment of Complement-Activated HUS

Eculizumab was approved to treat complement-activated HUS (aHUS) in both the USA and many European countries in 2011. It reduces anaphylatoxin-induced inflammation and limits the prothrombotic effects of C5b-9 complement activation. So far, three studies have been published, two in adolescents and adults and one in children and adolescents, that have shown the effectiveness of a 26-week treatment with aHUS once every 2 weeks, and data on the long-term extension after 2 years have confirmed these positive results.

Ravulizumab, a humanized monoclonal antibody that blocks terminal complement activation at C5, was created by modifying eculizumab. It has an extended half-life and can be administered once every 8 weeks. Its efficacy has been confirmed in adults, and a pediatric study has also showed the safety and efficacy of the drug given every 4–8 weeks at a dose based on body weight [16]. Ravulizumab was approved to treat aHUS in the USA in 2019. In acute phase a of HUS, the use of eculizumab is recommended, which can be safely changed to ravulizumab when the patient’s clinical status is stable.

The optimal duration of treatment with eculizumab is unknown. Generally, it is suggested that the dosing interval could be extended to 4–6 weeks, especially when no complement gene variant was detected. Large multicenter prospective studies, including biopsy-confirmed remission with different doses of eculizumab and ravulizumab, are essential to standardize the long-term treatment of aHUS in patients with rare complement gene variants who are at high risk of relapse.

A number of potential treatments for aHUS are currently being investigated. Krowalimab is an intravenous C5 receptor antagonist that recognizes epitopes on C5 other than eculizumab. Its use would allow the neutralization of C5 in patients with certain genetic polymorphisms that prevent the binding of eculizumab.

Avacopan is an orally administered C5aR1 antagonist that inhibits C3a, C4a, and C5a functions. It has already been shown to be effective in neutrophil cytoplasmic antibody (ANCA)-positive vasculitis. A phase II study in patients with aHUS on dialysis has been completed. Iptakopan (LNP023) is a first-in-class, orally administered, potent, and highly selective inhibitor of the alternative complement pathway factor B; however, clinical trials are currently underway in adults who have not previously been treated with complement inhibitors [17].

## 3. Idiopathic Nephrotic Syndrome

In 2022, PTNFDz also published updated recommendations for the management of children with nephrotic syndrome (NS) [18]. Among the significant changes, the recommendation to determine the dose and duration of steroid therapy in relapsed and steroid-dependent NS to achieve the desired effect at the minimum effective dose should be emphasized. Failure to achieve the desired effect, manifested by the persistence or increased frequency of relapses, is a sign for the extension of treatment with other drugs.

The recommendations suggest that during respiratory tract infections, the dose of glucocorticoids (GCs) should be increased, e.g., administered daily, if corticosteroids have been administered every 2 days so far, or that corticosteroids should be included for the period of infection, because in children with recurrent DS in remission and after therapy, this reduces the risk of relapse [14]. This suggestion was based on previous studies, conducted in small groups, which indicated that daily administration of low doses of prednisolone for 5–7 days during upper respiratory tract infections reduces the risk of relapse.

To confirm these observations, a double-blind placebo-controlled RCT (PREDNOS 2) was planned in 2021 to evaluate 365 children with recurrent steroid-sensitive FS conducted in 122 pediatric units in the UK. However, this study did not show that daily low doses of prednisolone for 6 days during upper respiratory tract infections reduced the risk of the recurrence of DS in children [19].

## 4. IgA Nephropathy in Children

In order to confirm the suspicion of IgA nephropathy in a child, as well as to assess inflammation, a kidney biopsy is recommended [20].

There are significant differences in histological changes between children and adults. In children, mesangial and endocapillary hypercellularity is usually observed. In adults, focal glomerulonesclerosis and tubular atrophy/interstitial fibrosis are more common. Hence, it is not recommended to use the International IgAN Prediction Tool, which was previously used for predicting IgAN in adults, for children [21]. Therefore, the International Pediatric IgAN Prediction Tool (https://qxmd.com/calculate/calculator_713/international-igan-prediction-tool-at-biopsy-pediatrics (accessed on 21 February 2025) was designed to predict IgAN in pediatric patients [22]. The pediatric tool allows us to predict the risk of a 30% decline in the estimated glomerular filtration rate (eGFR) or kidney failure in children at the time of biopsy, as well as the additional risk calculation one or two years after the biopsy [22].

For pediatric patients with IgAN, KDIGO recommends lifestyle interventions such as a low sodium diet and regular blood pressure control to keep the blood pressure below the 90th percentile for gender, age, and height in all pediatric patients. The first-line antihypertensive treatment in IgA nephropathy is RASB or calcium channel blockers [23]. If the proteinuria is above 0.2 g/day (or UPCR > 0.2 mg/mg), using RASB is recommended regardless of the blood pressure.

There are also new recommendations for the treatment of IgA nephropathy in children. KDIGO recommends corticosteroid treatment for pediatric patients with IgAN when proteinuria exceeds 1 g/day (or UPCR > 1 mg/mg) or when mesangial hypercellularity is observed [20]. A consensus on the usage of corticosteroids in pediatric IgA has not been achieved. The VALIGA study (European Validation of the Oxford Classification of IgA Nephropathy) recommends treatment with corticosteroids for patients without high-risk features [24]. Aside from corticosteroids, drugs recently studied for the treatment of IgAN include budesonide, hydroxychloroquine, and dapagliflozin; however, these were only pilot studies [25,26,27]. More research is needed to evaluate their use in the treatment of IgAN in children. There are many other therapies used to treat IgA nephropathy that are not extensively discussed in KDIGO 2021. Mycophenolate mofetil is most often referred to as a drug that may provide potential benefits to patients [28]. Cyclophosphamide is recommended only for rapidly progressive glomerulonephritis, although there is no powerful evidence in the literature to support its use. Other therapies, such as calcineurin inhibitors, are listed as drugs that have no proven benefit for patients with IgA nephropathy [29].

Children usually have more inflammation, mesangial hypercellularity, and capillary proliferation than adults do at biopsy and likely present earlier for macroscopic hematuria [30,31]. At the time of diagnosis, children have a higher GFR and less severe proteinuria than adults [32].

Most nephrologists recommend initiation of immunosuppressive therapy with glucocorticoids in cases with proteinuria > 1 g/day (or UPCR > 1 mg/mg) and/or mesangial hypercellularity [20]. Treatment usually includes 4 weeks of oral prednisolone at a dose of 1–2 mg/kg/day, followed by a gradual dose reduction every other day for another 4–6 months [20]. In some cases, steroid treatment is used for up to 24 months [33]. No specific recommendations are offered by KDIGO in this field [20].

## 5. Primary Hyperoxaluria

Primary hyperoxaluria (PH) is a family of rare genetic disorders of the liver in which enzyme deficiencies cause an accumulation of oxalate in the kidneys and other body organs. The overproduction of endogenous oxalate leads to recurrent kidney stones, nephrocalcinosis, and eventually kidney failure. Later, it can cause life-threatening systemic disease.

Until recently, PH1 treatment was supportive, burdensome for patients, and showed little effectiveness. Even adherence to hydration and citrate cannot prevent the development of kidney failure in patients with PH1. New therapies, particularly those based on RNAi, have shown promise in reducing the oxalate production in PH1 patients, at least in the short term. Emerging data on their clinical efficacy suggest that these drugs could indeed revolutionize PH1 treatment soon.

In response to the potential for promising therapies, the European Reference Network for Rare Kidney Diseases (ERKNet) working group on metabolism has been established.

## 6. Indications and Justification for the Use of RNAi Therapy

Patients with PH1 currently have access to two RNAi therapies. Lumasiran works by silencing the gene encoding the enzyme glycol oxidase, which catalyzes the conversion of glycol into glyoxylate. In the randomized controlled trial Illuminate A in 39 patients with PH1, patients > 6 years of age taking lumasiran showed an average reduction in urinary oxalate excretion of 65% compared with 11% in patients receiving a placebo. A decrease in urinary oxalate levels below 1.5 times the upper reference level after 6 months of treatment was observed in 84% of treated patients, and 52% had complete normalization of urinary oxalate excretion [34].

In an open-label study of Illuminate B in 18 children < 6 years of age, including infants, treatment with lumasiran resulted in a reduction in the urine sample oxalate to creatinine ratio in 72 and a reduction to 1.5 times the upper limit of normal in 50% of patients [35].

The second RNAi drug is nedosiran, which inhibits the production of l-lactate dehydrogenase A (LDHA), which is necessary for the cytosolic conversion of glyoxylate into oxalate. In a randomized PHYOX 2 study that included patients with PH1 or PH2 and an eGFR of >30 mL/min/1.73 m^2^, there was a 59% reduction in urinary oxalate concentrations in patients with PH1, but there were no reductions in PH2 patients [36].

Currently, there are no long-term studies to assess whether lumasiran and nedosiran improve outcomes in patients with PH1, in particular, whether they affect the recurrence of nephrolithiasis, the regression of nephrocalcinosis, and most importantly, further deterioration in kidney function.

Research is currently underway on the use of stiripentol, an oral drug used for genetic epileptic encephalopathy (Dravet syndrome). The low urinary oxalate concentration after use of stiripentol contributed to the initiation of studies to determine the efficacy of stiripentol as monotherapy in patients > 6 months of age with PH1–3 and eGFR > 45 mL/min/1.73 m^2^ [37]. In this editorial, we highlight the most up-to-date guidelines and recommendations for the management of kidney diseases in children.

## 7. Highlights

- Urinalysis screening for asymptomatic bacteriuria, as well as its treatment, is not recommended in patients with a catheter remaining in the bladder for up to 30 days.
- A urine culture should be performed before a potentially traumatizing instrumental procedure within the urinary tract, and if asymptomatic bacteriuria is found, two doses of a targeted antibiotic should be administered: before and after the procedure.
- Antibiotic therapy is not recommended in patients with a urine culture of *Pseudomonas aeruginosa* unless there are clinical signs of UTI. Bacteriuria and pyuria, without UTI symptoms, are not an indication for treatment. Treatment should apply to patients who are expecting an instrumental urinary tract procedure or surgery.
- The decision to use antibacterial prophylaxis should be made by a pediatric nephrologist after exhausting non-pharmacological methods. The decision on chronic prevention should be reviewed every 6 months.
- It is recommended to use pharmacological prophylaxis for UTI in children with stage III–V VUR.
- Routine antimicrobial prophylaxis is not recommended to prevent catheter-related infections, either with a single catheterization or if the catheter is left in the bladder for a long time.
- There is no simple answer as to whether every child with a febrile UTI should undergo an ultrasound.
- Daily administration of low doses of corticosteroids to children with recurrent corticotic syndrome during upper respiratory tract infections does not prevent a recurrence associated with the upper respiratory tract infection.
- Using eculizumab to treat STEC-HUS is not recommended.
- In acute phase a of HUS, the use of eculizumab is recommended, which can be safely changed to ravulizumab when the patient’s clinical status is stable.
- The optimal duration of treatment with eculizumab is not known.
- The International Pediatric IgAN Prediction Tool (https://qxmd.com/calculate/calculator_713/international-igan-prediction-tool-at-biopsy-pediatrics, accessed on 21 February 2025) was created to predict IgAN in pediatric patients.
- In children with IgAN, KDIGO recommends the use of RASB in all patients with proteinuria > 0.2 g/day (or UPCR > 0.2 mg/mg), regardless of the blood pressure values.
- KDIGO recommends corticosteroid treatment for pediatric patients with IgAN in cases of proteinuria > 1 g/day (or UPCR > 1 mg/mg) or mesangial hypercellularity.
- Drugs recently studied for the treatment of IgAN include budesonide, hydroxychloroquine, and dapagliflozin.
- In response to the potential for promising therapies in rare diseases, including hyperoxaluria, the European Reference Network for Rare Kidney Diseases (ERKNet) Working Group on Metabolism was established.

This editorial summarizes the most up-to-date guidelines and recommendations for management of kidney diseases in children. In recent years, significant progress has been made in pediatric nephrology, which covers many aspects, such as diagnosis, treatment, and the principles for the management of the most common kidney diseases in children.

## Abbreviations

HUS—hemolytic–uremic syndrome, VUR—vesicoureteral reflux, PH—primary hyperoxaluria, US—ultrasound examination, STEC-HUS—HUS associated with infection with the *Escherichia coli* bacterium producing Shiga toxin, NS—nephrotic syndrome, UTI—urinary tract infection, PTNFDz—Polish Society of Pediatric Nephrology, IgAN—IgA nephropathy.
